# Dissolved polycyclic aromatic compounds in Canada’s Athabasca River in relation to Oil Sands from 2013 through 2019

**DOI:** 10.1007/s10661-023-11846-x

**Published:** 2023-10-21

**Authors:** Lucie M. J. Lévesque, Julie Roy, Nancy E. Glozier, Leah Dirk, Colin A. Cooke

**Affiliations:** 1 Environment and Climate Change Canada, Saskatoon, Saskatchewan S7N 3H5 Canada; 2 Environment and Parks, Government of Alberta, Edmonton, Alberta T5J 5C6 Canada; 3 Earth and Atmospheric Sciences, University of Alberta, Edmonton, Alberta T6G 2E3 Canada

**Keywords:** Oil sands, Water, Monitoring, Alkylated polycyclic aromatic compounds

## Abstract

The Canada-Alberta Oil Sands Monitoring (OSM) Program began long-term surface water quality monitoring on the lower Athabasca River in 2012. Sampling of low level, bio-accumulative polycyclic aromatic compounds (PACs) targeted a suite of parent and alkylated compounds in the Athabasca River (AR) mainstem using semi-permeable membrane devices (SPMDs). Samples were collected along a gradient from upstream reference near Athabasca, Alberta, through exposure to the Athabasca oil sands deposit (AOSD), various tributary inflows, and mining activities within the OSMA, to downstream recovery near Wood Buffalo National Park (WBNP) and reference on the Slave River. The program adapted over the years, shifting in response to program review and environmental events. The AOSD chemical fingerprint was present in samples collected within the AOSD, through the oil sands mineable area (OSMA), downstream to recovery from 2013 to 2019. PACs were dominated by alkylated phenanthrenes/anthracenes (PAs) and dibenzothiophenes (Ds), with elevated levels of alkylated fluorenes (Fs), naphthalenes (Ns), fluoranthenes/pyrenes (FlPys) and benzo[a]anthracenes/chrysenes (BaACs), increasing in concentration from C1 < C2 < C3 < C4. Concentrations of these petrogenic PACs were at their highest within the OSMA and downstream of tributaries. The AOSD fingerprint was absent from sites located outside of the influence of the AOSD and downstream of the Peace-Athabasca Delta on the Slave River. PAC concentrations in the AR increased with mainstem discharge and loadings from tributaries, were moderated by the PAD, and diluted by the Peace River. This work bolsters the baseline PAC information previously reported for the Athabasca River and waters downstream, reporting 7 years of data, from all sites within the mainstem monitoring program, and exploring potential regional and hydrological drivers of these between sites and over time.

**Supplementary Information:**

The online version contains supplementary material available at 10.1007/s10661-023-11846-x.

## Introduction

The governments of Canada and Alberta designed an environmental monitoring program to determine the state of environment and assess cumulative effects of oil sands development in Northeastern Alberta, Canada (Environment Canada and Alberta Environment, 2011a, 2011b). The lower Athabasca River (AR) surface water quality monitoring program was part of the Oil Sands Monitoring (OSM) Program implemented in 2012 (Environment Canada and Alberta Environment, 2012). The objectives of the OSM Program included assessing the current state of water quality and the distribution of contaminants in surface waters of the Athabasca (mainstem), Peace and Slave rivers and riverine waterbodies within the Peace-Athabasca Delta (PAD; Glozier et al., 2018). In addition to providing information on contemporary baseline, the program aimed to facilitate assessment of natural and anthropogenic compounds within these waterbodies. The surface water quality component of the OSM Program was reviewed and revised in 2017 (Cooke et al., 2018). Based on the initial results of this review, the optimized design included use of passive sampling of dissolved PACs at key sites in the mainstem reaches of the Athabasca, Peace and Slave rivers, an approach that was also implemented as a measure of exposure in OSM biomonitoring programs (Culp et al., 2018, 2020).

Dissolved potentially toxic, bio-accumulative polycyclic aromatic compounds (PACs) occur naturally within the Athabasca oil sands deposit and are present in waterbodies within and downstream of the oil sands region (Conly et al., 2002; Evans et al., 2002; Hall et al., 2012; Headley et al., 2001). These compounds have the potential to be elevated in concentration above those occurring naturally, persisting in the environment and posing risks to biota (CCME, 2010). Oil sands development and mining operations, including landscape-induced changes to runoff and erosion, combustion, bitumen extraction and processing, and leakage from tailings ponds, have the potential to contribute PACs in both naturally-occurring and potentially more toxic forms (Culp et al., 2021; Kelly et al., 2009; Manzano et al., 2016; Mundy et al., 2019; Yang et al., 2011). The objectives of the OSM surface water quality passive sampling program were to determine current concentrations of dissolved PACs, their distribution through the monitoring area, and potential sources of the compounds (Glozier et al., 2018).

Glozier et al. (2018) evaluated and interpreted data from the surface water quality monitoring component over the three-year implementation phase (2012–2015). This monitoring data set was the most spatially- and temporally-intensive in the history of the oil sands region, providing an initial characterization of the status and patterns of surface water quality as a contemporary baseline. The results indicated that semi-permeable membrane devices (SPMDs) used to passively sample PACs effectively detected trace-level unsubstituted PACs (i.e., parent compounds such as the 16 United States (US) Environmental Protection Agency (EPA) priority pollutants (PP) commonly assessed as threats to aquatic ecosystems), as well as an extensive suite of abundant, substituted PACs (i.e., alkylated compounds of toxic potential; Andersson & Achten, 2015). The magnitudes and spatial distributions of PAC concentrations and their relative abundances varied with season and sampling location relative to the Athabasca oil sands deposit (AOSD), tributary inflows, and the PAD. In this paper, we present and extend analysis of the three years previously reported by Glozier et al. (2018) to include SPMD monitoring results for dissolved PACs from 2013 through to and including 2019, examining chemical fingerprints, temporal and spatial variations in PAC concentrations over the seven years, and relationships with discharge.

## Materials and methods

### Monitoring design

**Fig. 1:**
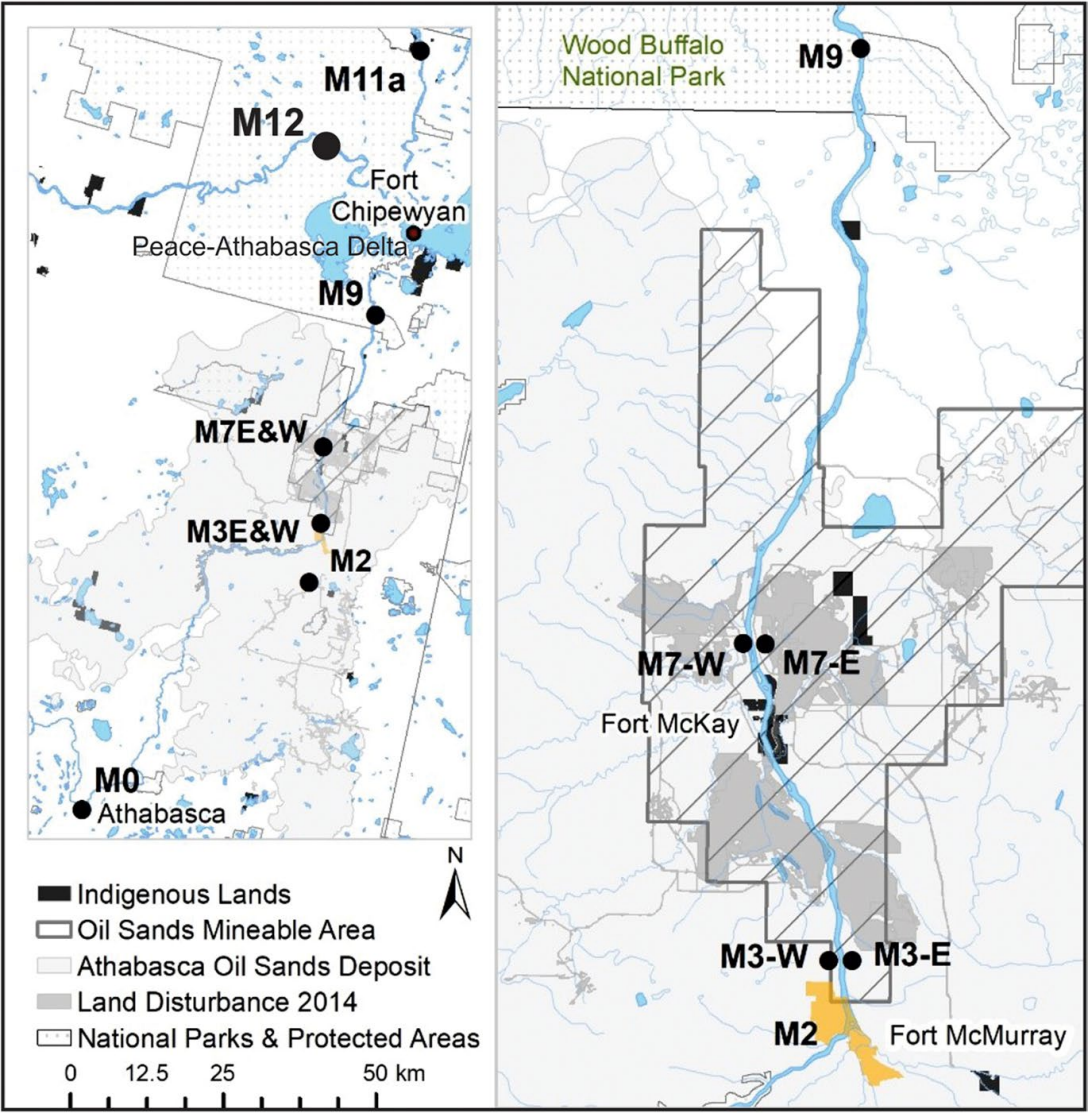
Monitoring area

The design of the OSM long-term surface water quality monitoring program is detailed in the joint Canada-Alberta monitoring, integration and implementation plans (Environment Canada and Alberta Environment, 2011a, 2011b, 2012). SPMDs were deployed on a trial basis as part of program implementation in 2011 and 2012. In 2013 they became a routine part of the long-term surface water quality monitoring component of OSM, providing a high frequency of PAC detection relative to water quality grab samples (Glozier et al., 2018). Mainstem sampling sites were located along the Athabasca River (AR) between the town of Athabasca within the Middle Athabasca Region (MAR), through the Lower Athabasca Region (LAR) within the bitumen-rich AOSD and oil sands mineable area (OSMA), downstream toward Wood Buffalo National Park (WBNP) and the Peace-Athabasca Delta (PAD), to the Slave River (SR). This monitoring design represented a gradient in watershed conditions and potential exposures to natural and anthropogenic PACs (Fig. 1).

Reference sampling sites, those well outside of the AOSD, were located at the upstream (southern) and downstream (northern) extents of the monitoring area. The upstream-most site (M0) was located within the middle Athabasca region on the mainstem near the town of Athabasca, and the downstream-most site (M11a) was on the Slave River near Fort Fitzgerald, downstream of the PAD. Mainstem site M9, located within the lower Athabasca region near the border of WBNP, was identified as a recovery site, located approximately 85 km downstream of the AOSD. Sampling on the Peace River (PR) at peace point (M12; see supplementary information (SI)) was conducted to support interpretation of PAC concentrations in the SR (M11a), downstream of the PAD and confluence with the PR.

Sampling sites within the AOSD were located along the AR mainstem upstream and downstream of areas affected by anthropogenic activities and tributaries. Site M2 was located within the AOSD, upstream of the city of Fort McMurray and the OSMA. The remaining sampling sites were located within the OSMA: M3 downstream of Fort McMurray, upstream of oil sands-related land disturbance (Fig. 1), toward the AR west (M3W) and east (M3E, downstream of Clearwater River) banks; M4 upstream of Fort McKay and downstream of the Steepbank River; and, M7 near the northern-most extent of oil sands activity, downstream of MacKay river, toward the AR west (M7W) and east (M7E, downstream of Ells River) banks.

### Sample collection

During the first five-year cycle of the OSM surface water quality monitoring program, passive sampling was adapted as methods were refined and program priorities shifted. The success of sample retrieval increased over this time as the anchoring system for the deployment arrays was improved (ECCC, 2018a), reducing sample losses with shifting sand beds and debris-laden flows. Starting in 2013 sampling took place during both the open-water and under-ice seasons at M0, M2, M4, M7, M9 and M11a (Table 1, SI1; Environment Canada and Alberta Environment, 2011b). In 2014 and 2015 the program added M3E and M3W as sampling sites on either side of the AR channel, downstream of Fort McMurray and upstream of mining activities. In 2015, to allow for a balanced design within the OMSA, M7E and M7W were added downstream of mining activities. SPMD sampling was temporarily scaled back in 2016, with collections at upstream reference (M0), within the AOSD upstream and downstream of Fort McMurray (M2, M3), and at downstream recovery (M9), during the open-water season. Canada’s historic Horse River Wildfire swept through the region that same year, resulting in emergency evacuation of nearly 90,000 residents of Fort McMurray and burning 590,000 ha of land. In response, an intensive mainstem and tributary monitoring program was implemented by the province of Alberta (Emmerton et al., 2020). ECCC scaled up sampling to include tributaries within and downstream of the burn area (unpublished). After the 2016 fire, the core long-term SPMD sampling sites and frequencies stabilized.

**Table 1:** Samples collected from the Middle Athabasca Region, Lower Athabasca Region and SR from 2013 to 2019

YEAR	MAR	LAR			SR
		Upstream of OSMA	Within OSMA	Downstream of OSMA	
2013	M0 ^(1)^	M2 ^(2)^	M4 ^(4)^ M7E ^(4)^	M9 ^(4)^	M11a ^(5)^
2014	M0 ^(2, 4)^	M2 ^(2, 4)^	M3E ^(1, 5)^, M3W ^(1, 5)^ M4 ^(2, 3)^ M7E ^(4)^	M9 ^(1, 3)^	M11a ^(1, 5)^
2015	n/s	M2 ^(1, 3)^	M3E ^(1, 3)^, M3W ^(1, 3)^ M4 ^(1)^ M7E ^(1, 3)^, M7W ^(3)^	M9 ^(2)^	M11a ^(1, 4)^
2016	M0 ^(4)^	M2 ^(3)^	M3E ^(2)^, M3W ^(3)^	M9 ^(4)^	n/s
2017	n/s	M2 ^(5)^	M3E ^(5)^, M3W ^(3)^ M7E ^(4)^, M7W ^(5)^	M9 ^(3)^	n/s
2018	n/s	M2 ^(5)^	M3E ^(5)^, M3W ^(5)^ M7E ^(5)^, M7W ^(5)^	M9 ^(5)^	n/s
2019	n/s	M2 ^(5)^	M3E ^(1)^, M3W ^(5)^ M7E ^(5)^, M7W ^(1)^	M9 ^(5)^	n/s

n/s = no samples; (N, N) = number of under-ice, open-water samples; (N) = number of open-water samples; E = toward east bank; W = toward west bank.

SPMDs were deployed and retrieved monthly (Environment and Climate Change Canada, 2018a, 2018b). These devices, which mimic biological uptake of compounds, were fabricated by Environmental Sampling Technologies (EST https://www.est-lab.com/). All membranes, including blanks, were spiked with performance reference compounds (PRCs; 10 μg Fluoranthene-d10, 10 μg Anthracene-d10 per membrane), to allow for calibration of in-situ rates of PAC exchange between the membranes and the environment (Alvarez, 2010). The membranes were placed in a canister that was suspended at approximately 0.5 m depth from an anchored deployment array. Arrays were placed within the thalweg at each site or at the deepest locations on either side of the river for multiple east-west deployments. Every sample was accompanied by a field blank that was exposed to the air alongside the sample at deployment and retrieval, accompanying the sample during all time outside of the water. Quality assurance quality control (QAQC) sampling events included replicate samples and a trip blank (Environment and Climate Change Canada, 2018c). Upon retrieval, samples and blanks were left in cold storage until submitted for chemical analysis.

### Chemical analyses

Samples were submitted to SGS AXYS (https://www.sgsaxys.com/) for analysis of parent and alkylated PACs (Table 2, Table SI2). Membranes were inspected, cleaned, and rinsed with hydrochloric acid, after which they were dialyzed twice in hexane following AXYS Standard Operating Procedure (SOP) SLA-188 (AXYS Analytical Services Ltd., 2014). The hexane extracts were then spiked with isotopically-labeled PAC surrogate standards and concentrated using rotary evaporation. As per AXYS Method MLA-021 (AXYS Analytical Services Ltd., 2013; based on the EPA Method 8270C/D modified by EPA 1625B Semivolatile Organic Compounds by Isotope Dilution GC/MS), extracts were cleaned using column chromatography on silica and analyzed using GC/MS (high resolution Gas Chromatography coupled with quadrupole Mass Spectrometry) with data acquisition in SIM (Selected Ion Monitoring) mode. Quantification of internal standards was used for recovery correction of analytical results.

**Table 2:** Dissolved PAC analyte list

2-ring carbon		4-ring carbon	
Biphenyl	B	Fluoranthene	Fl
C1-Biphenyls	C1-B	Pyrene	Py
C1-Naphthalenes	C1-N	C1-Fluoranthenes/Pyrenes	C1-FlPy
C2-Biphenyls	C2-B	C2-Fluoranthenes/Pyrenes	C2-FlPy
Naphthalene	N	C3-Fluoranthenes/Pyrenes	C3-FlPy
C2-Naphthalenes	C2-N	C4-Fluoranthenes/Pyrenes	C4-FlPy
C3-Naphthalenes	C3-N	Benz[a]anthracene	BaA
C4-Naphthalenes	C4-N	Chrysene	C
3-ring carbon		C1-Benzo[a]anthracenes/Chrysenes	C1-BaAC
Acenaphthene	Ac	C2-Benzo[a]anthracenes/Chrysenes	C2-BaAC
C1-Acenaphthenes	C1-Ac	C3-Benzo[a]anthracenes/Chrysenes	C3-BaAC
Acenaphthylene	Acl	C4-Benzo[a]anthracenes/Chrysenes	C4-BaAC
Fluorene	F	5-ring carbon	
C1-Fluorenes	C1-F	Benzo[b]fluoranthene	BbFl
C2-Fluorenes	C2-F	Benzo[j,k]fluoranthenes	BjkFl
C3-Fluorenes	C3-F	Benzo[a]pyrene	BaPy
Phenanthrene	P	Benzo[e]pyrene	BePy
Anthracene	A	C1-Benzofluoranthenes/Benzopyrenes	C1-BFlBPy
C1-Phenanthrenes/Anthracenes	C1-PA	C2-Benzofluoranthenes/Benzopyrenes	C2-BFlBPy
C2-Phenanthrenes/Anthracenes	C2-PA	Dibenz[a,h]anthracene	DahA
C3-Phenanthrenes/Anthracenes	C3-PA	Perylene	Pe
C4-Phenanthrenes/Anthracenes	C4-PA	6-ring carbon	
Retene	R	Indeno[1,2,3-cd]pyrene	IcdPy
3-ring sulfur		Benzo[g,h,i]perylene	BghiPe
Dibenzothiophene	D		
C1-Dibenzothiophenes	C1-D		
C2-Dibenzothiophenes	C2-D		
C3-Dibenzothiophenes	C3-D		
C4-Dibenzothiophenes	C4-D		

### Data analyses

Prior to analysis, the raw data underwent quality assurance checks, background-correction, and conversion to estimated time-weighted dissolved concentrations (ng/L). The data were treated as per ECCC SOPs (Environment and Climate Change Canada, 2018c) and concentrations were calculated using the United States Geological Survey (USGS) SPMD-Water Concentration Estimator-v5. Censored data (i.e., values below analytical detection or below background as defined by field blanks) were replaced with 0.5 times their sample-specific reporting limits. Microsoft Excel was used to calculate summary metrics including total (TPAC, the sum of all analytes listed in Table 2), alkylated (APAC, the sum of all alkylated PACs in Table 2) and parent (PPAC, the sum of all parent PACs in Table 2) PACs, and to produce chemical profiles (fingerprints) using the mean and standard error of individual PAC concentrations (Boehm & Saba, 2008; Stogiannidis & Laane, 2015; Yang et al., 2011).

Boxplots of PAC concentrations were generated using SigmaPlot v.12.5, displaying range and variability in PAC concentrations within regions, over years, and between sites. Spearman Rank Order correlations between PACs and cumulative discharge (CQ) were used to examine hydrology as a potential driver of PAC concentrations. CQ was calculated using daily discharges from Water Survey of Canada WSC (https://wateroffice.ec.gc.ca/) stations located in closest proximity to each sampling site, over each deployment period; discharges for M2 were calculated by subtracting the Clearwater at Draper station (07CD001) from the Athabasca downstream of Fort McMurray station (07DA001). The pyrogenic index (PI), calculated as per Wang et al. (1999; Eq. PI), provided an initial look at petrogenic (PI <0.8, crude <0.01, heavy oils/fuels <0.05) and pyrogenic (PI 0.8–2.0) PACs (Stogiannidis & Laane, 2015; Wang et al., 1999).

$$\mathrm{Eq}.\mathrm{PI}=\frac{\sum\left(\mathrm{acenaphthylene},\;\mathrm{acenaphthene},\;\mathrm{anthracene},\;\mathrm{fluoranthene},\;\mathrm{benz}\left(\mathrm a\right)\mathrm{anthracene},\;\mathrm{pyrene},\;\mathrm{benzo}\left(\mathrm b\right)\mathrm{fluoranthene},\;\mathrm{benzo}\left(\mathrm k\right)\mathrm{fluoranthene},\;\mathrm{benzo}\left(\mathrm e\right)\mathrm{pyrene},\;\mathrm{benzo}\left(\mathrm a\right)\mathrm{pyrene},\;\mathrm{perylene},\;\mathrm{dibenz}\left(\mathrm{ah}\right)\mathrm{anthracene},\;\mathrm{indeno}\left(1,2,3-\mathrm{cd}\right)\mathrm{pyrene},\;\mathrm{benzo}\left(\mathrm{ghi}\right)\mathrm{perylene}\right)\;}{\sum\left(\mathrm C0-\mathrm C4\;\mathrm{naphthalenes},\;\mathrm C0-\mathrm C4\;\mathrm{phenanthrenes}/\mathrm{anthracenes},\;\mathrm C0-\mathrm C3\;\mathrm{fluorenes},\;\mathrm C0-\mathrm C3\;\mathrm{dibenzothiophenes},\;\mathrm C0-\mathrm C3\;\mathrm{benzo}\left(\mathrm a\right)\mathrm{anthracenes}/\mathrm{chrysenes}\right)}$$

Principal Components Analyses (PCA) were completed using Primer 7, including all PACs at every sampling site to determine the degree of similarity in concentrations between sites. A total of 146 samples from 9 sites were analyzed. Boxplots for the most abundant and frequently detected PACs were examined to further assessment of PAC concentrations along the AR mainstem.

## Results

### Regional concentrations

PAC concentrations within the monitoring area were highest in the Lower Athabasca Region within and downstream of the OSMA (M3-M7 and M9; Table 3). Mean TPACs increased by over 10-fold from reference 4.66 ± 0.50 ng/L at upstream reference (M0) to 52.80 ± 4.03 within the OSMA (M3-M7). Mean concentration dropped by approximately 20% downstream at M9 (41.62 ± 4.16 ng/L) near WBNP, with a further 65% reduction in concentration at the downstream reference on the SR (M11a, 7.78 ± 0.83 ng/L). This reduction may be expected given the moderating effects of the PAD on water quality (Evans et al., 2002; Hall et al., 2012) and dilution by the PR. The PR had mean TPAC concentrations of 4.51 ± 0.62 ng/L (Table SI3), similar in magnitude and range to those measured at M0. The frequencies of PAC detections at the SR were slightly lower (55–90% per sample) than those at sites further upstream (61–100% per sample).

**Table 3:** PAC concentrations in the Middle Athabasca Region, Lower Athabasca Region upstream of (M2), within (M3-M7), and downstream of (M9) the OSMA, and the SR from 2013 to 2019

Region within Monitoring Area	Middle Athabasca Region (MAR)		Lower Athabasca Region (LAR)						Slave River (SR)	
Site ^(N)^	M0 ^(11)^		M2 ^(30)^		M3-M7^(101)^		M9^(27)^		M11a^(16)^	
PAC Subset	*Mean ng/L*	*SE*	*Mean ng/L*	*SE*	*Mean ng/L*	*SE*	*Mean ng/L*	*SE*	*Mean ng/L*	*SE*
∑PACs (TPAC)	4.66	0.50	19.98	1.49	52.80	4.03	41.62	4.16	7.78	0.83
∑Parent (PPAC)	1.17	0.08	1.80	0.16	3.27	0.17	3.10	0.23	1.31	0.23
∑Alkylated (APAC)	3.49	0.44	18.18	1.37	49.53	3.90	38.51	3.98	6.46	0.67
∑EPA 16 PP	1.02	0.05	1.56	0.14	2.86	0.15	2.67	0.22	1.07	0.20
∑CCME	0.09	0.01	0.19	0.01	0.57	0.03	0.61	0.03	0.17	0.02
∑C2	0.45	0.08	2.51	0.32	5.48	0.58	3.62	0.86	1.15	0.26
∑C3	2.83	0.26	10.01	0.83	22.87	1.73	16.45	1.69	3.60	0.45
∑S3	0.16	0.05	4.60	0.38	14.77	1.33	11.19	1.43	0.87	0.15
∑C4	1.05	0.18	2.59	0.16	9.10	0.59	9.71	0.65	1.89	0.17
∑C5	0.16	0.04	0.25	0.03	0.54	0.03	0.61	0.03	0.25	0.03
∑C6	0.01	0.00	0.01	0.00	0.02	0.00	0.03	0.00	0.01	0.00

SE = standard error; N = number of samples; TPAC = total PACs (sum of 49 analytes) per sample; PPAC = parent (unsubstituted) PACs; APAC = alkylated (substituted) PACs; EPA PP = (United States) Environmental Protection Agency Priority Pollutants; CCME = Canadian Council of Ministers of the Environment carcinogenic PACs.

The unsubstituted PACs (PPACs), most of which are US EPA Priority Pollutants (PP), were relatively uniform in concentration across regions as compared to the more abundant and variable alkylated compounds (APACs). Mean PPAC concentration peaked at 3.27 ± 0.17 ng/L in the OSMA, compared to 49.53 ± 3.90 ng/L for APACs. Samples from reference sites (M0, SR) contained mean PPACs at concentrations more than 60% reduced from that within the OSMA, with mean APACs 93% lower. The predominance of APACs within the Lower Athabasca Region is consistent with the characteristically elevated occurrence of alkylated homologs within the AOSD (Yang et al., 2011). APACs were above 77% detection in all samples for all regions, whereas PPACs were as low as 32% per sample (SR).

The relative magnitudes of PAC concentrations between sampling sites (M0 < M2 < M3-M7 > M9 > > M11a) were similar across most groups of PACs (Table 3), particularly the C3 and S3 compounds. This is typical of naturally-occurring, petrogenic PACs (Boehm & Saba, 2008; Jautzy et al., 2015; Yang et al., 2011). The nearly 28-fold increase in mean S3 concentration from M0 to M2 is consistent with the high abundance of polycyclic aromatic sulfur heterocyclic compounds that would be expected of samples collected from within the AOSD (Yang et al., 2011). Concentrations of C5 and C6 compounds were lower than other homologs, more frequently occurring below detection and at times all were absent from samples.

### Relative abundance

**Fig. 2:**
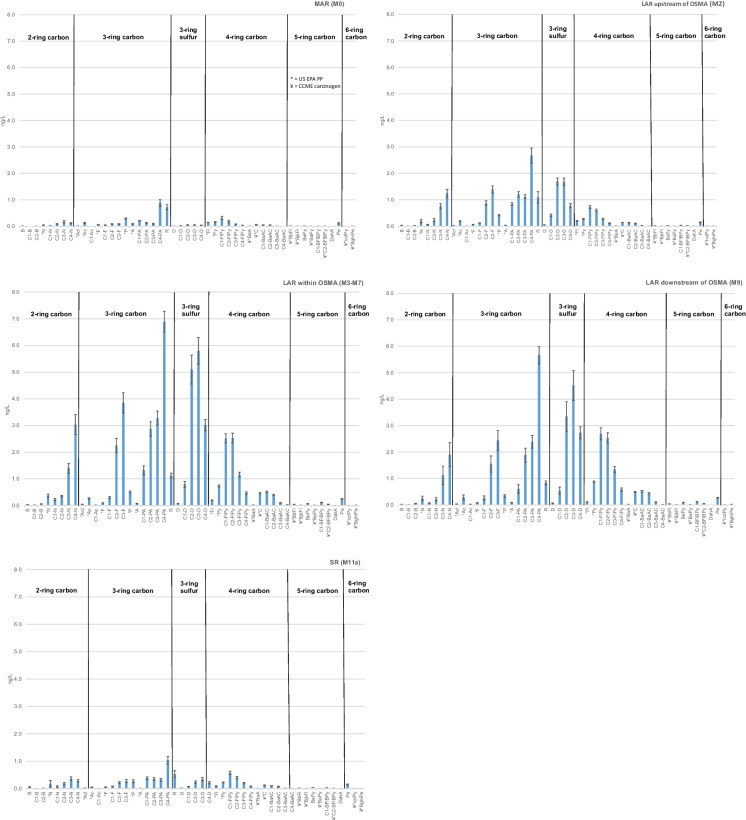
Mean 2013–2019 relative abundances of PACs across the monitoring area, from upstream reference (M0), through the OSMA (M3-M7), to the recovery location near the PAD (M9) and downstream reference (SR)

Mean relative abundances of PACs through the Lower Athabasca Region (M2, M3-M7, M9) from 2013 to 2019 exhibited the AOSD fingerprint. Samples had relatively high abundances of alkylated phenanthrenes/anthracenes (PAs), dibenzothiophenes (Ds), naphthalenes (Ns), fluorenes (Fs), fluoranthenes/pyrenes (FlPys), and benzo[a]anthracenes/chrysenes (BaACs) (Fig. 2; Wang et al., 2014, Yang et al., 2011). Parent compounds such as N, acenaphthylene (Acl), acenaphthene (Ac), F, P, A and Fl were present at magnitudes well below their alkylated forms, and were featured less prominently in these profiles than in those from outside of, and well downstream of, the AOSD (Middle Athabasca Region, SR, PR). Mean concentrations of pyrene (Py) and chrysene (C) exhibited little difference between regions, as did those of the five and six benzenoid ring priority pollutants and carcinogens (benzo[b]fluoranthene BbFl, benzo[j,k]fluoranthene BjkFl, benzo[e]pyrene BePy, benzo[a]pyrene BaPy, benzofluoranthenes/ benzopyrenes BFlBPys, dibenz[a,h]anthracene DahA, indeno[1,2,3-c,d]pyrene IcdPy, benzo[g,h,i]perylene BghiPe).

Alkylated PAs were typically the most abundant compounds in all profiles within the monitoring area. Mean concentrations within the AOSD and OSMA (M2, M3-M7), and downstream toward WBNP (M9), increased from C1 < C2 < C3 < C4. This AOSD-distinctive pattern of abundance in alkylated homologs was absent from reference sites, with mean concentrations of PAs decreasing from C1 through C3 upstream within the Middle Athabasca Region (M0) and remaining relatively uniform in magnitude downstream within the SR (Fig. 2). P and its alkyl-substituted compound retene (R), the latter of which has been used as an indicator of forest fire (Muir & Galarneau, 2021; Ramdahl, 1983), were more prominent in profiles from reference sites (M0, SR) than those within the Lower Athabasca Region.

Similar to alkylated PAs, Ds were highly abundant at sites within (M3-M7) and downstream (M9) of the AOSD. Mean concentrations of C2-C4 were greater than C0 (D) and C1 at all sites within the monitoring area, with C3-Ds pronounced in these profiles. Relative abundances of C2-C4s were notably low upstream of the AOSD at M0 and outside of the Athabasca mainstem within the PR (M12; Fig. SI1), indicating an absence of the AOSD fingerprint at these sites. Downstream of the confluence of the AR and PR, concentrations of alkylated Ds were depressed, indicating a reduction in these AOSD-characteristic compounds.

Alkylated PAs, Ds, Ns and Fs within and downstream of the Lower Athabasca Region typically increased in relative abundance from C1 to C3. This AOSD-characteristic pattern, which was identified by Yang et al. (2011) and Wang et al. (2014) in oil sands and river sediments within the lower AR watershed, was absent from other alkylated compounds (FlPys and BaACs). Mean concentrations of these homologs were relatively stable or decreased in concentration from C1-C4, similar to those at reference sites (M0, SR).

### Interannual variation

**Fig. 3:**
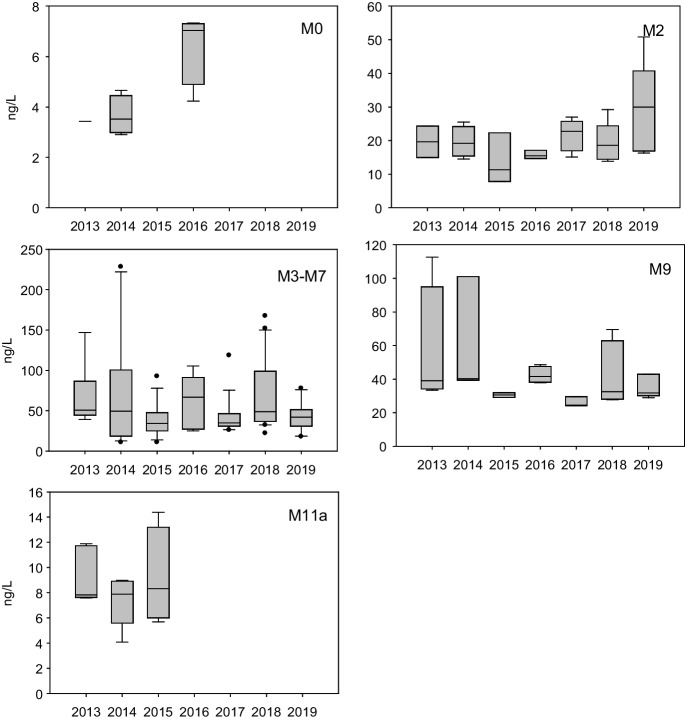
TPAC concentrations during open-water season in the Middle Athabasca Region (M0), Lower Athabasca Region (M2, M3-M7, M9) and the SR (M11a) from 2013 to 2019. Median plotted with 10th, 25th, 75th, and 90th percentiles

PACs were sampled every year during the open-water season. Annual median TPAC concentrations (Fig. 3) were between 3.5 and 7.0 ng/L at the upstream-extent of the monitoring area (M0), and 7.8 to 8.3 ng/L at the downstream extent (M11a). Within the AOSD upstream of the OSMA (M2) median TPAC concentrations were lowest in 2015 at 11.3 ng/L and highest in 2019 at 30 ng/L. Concentrations within M3-M7 were the most variable of all regions, ranging from 10.8 to 228 ng/L. Median concentrations were between 34.1 ng/L (2015) and 66.8 ng/L (2016), the latter occurring during scaling back of the program and sampling in response to the Horse River wildfire. Near the boundary of WBNP (M9) TPAC concentrations were as high as 113 ng/L (2013), with medians between 24.5 and 41.6 ng/L. Inter-annual patterns for TPAC concentrations generally held for parent and alkylated compounds (fig. SI2), with APAC concentrations orders of magnitude greater than PPACs within the lower Athabasca region (M2, M3-M7, M9). Annual data were insufficient to evaluate trends over time.

Under-ice samples were collected in the early part of the monitoring program (Table 1). TPAC concentrations were consistently lower than those measured during open-water at all sites (Fig. SI2-i) with PAC detections at times reaching <60% per samples (2 samples at M11a; Table SI3). Median concentrations were 2.2 and 3.8 ng/L at reference sites (M0 and M11a, respectively), 14.4 ng/L upstream of the OSMA (M2), 17.2 ng/L within the OSMA(M3-M7), and 16.8 ng/L downstream of the OSMA (M9). The highest under-ice TPAC concentration was 28.0 ng/L (M3-M7) nearly 10-fold lower than that in open-water (228 ng/L M3-M7).

### Pyrogenic indices

**Fig. 4:**
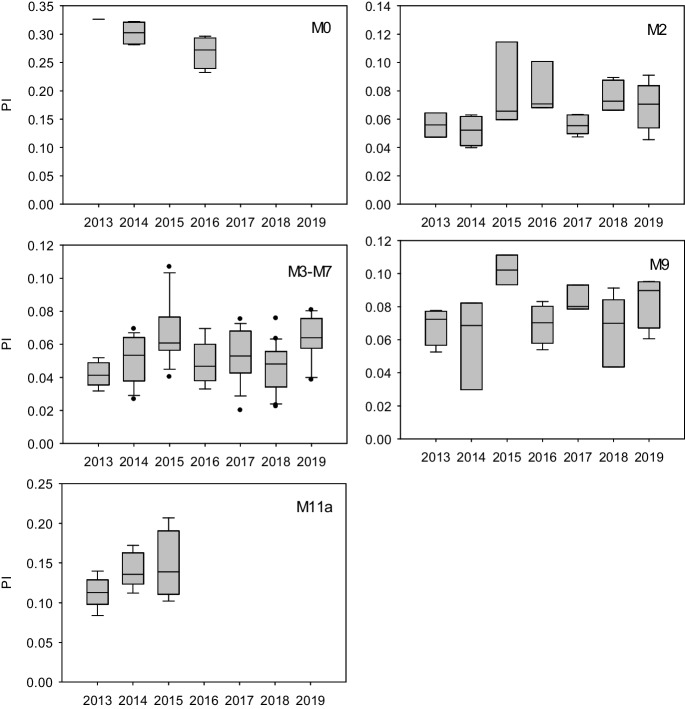
PIs during open-water season in the Middle Athabasca Region (M0), Lower Athabasca Region (M2, M3-M7, M9) and the SR (M11a) from 2013 to 2019. Median plotted with 10th, 25th, 75th, and 90th percentiles

The PIs at all sites within the monitoring area were consistent with PACs of petrogenic origin (<0.8; Stogiannidis & Laane, 2015; Wang et al., 1999). Reference site PIs were slightly higher than those within the Lower Athabasca Region, ranging between 0.23–0.33 upstream within the Middle Athabasca Region, and 0.084–0.21 downstream within the SR (Fig. 4). Within the Lower Athabasca Region PIs were typically below 0.1, consistent with the higher relative abundance of APACs at M2, M3-M7 and M9 (Table 3, Fig. SI2). There was no indication of an effect of the Horse River Wildfire on the mainstem PIs in 2016 or following years. Cooke et al. (2022) characterized PACs in ash from the wildfire, bitumen outcrops, and river water (before and after the fire); petrogenic compounds dominated postfire, with short episodic pyrogenic signatures in burned watersheds. Emmerton et al. (2020) found that although the wildfire affected water quality (suspended sediments, nutrients, metals) in small sub-watersheds in the vicinity of the fire, these local effects did not translate to the AR mainstem; the effects of disturbance were buffered by concentration-dilution associated with the large, low relief of the watershed. Concentrations of R (Fig. SI3) also show no indication of effect of the 2016 wildfire on water quality in the Lower Athabasca Region.

### Hydrology

PAC concentrations were significantly correlated with CQ at most sites within the monitoring area (Table SI4). The strongest associations between discharge and TPACs occurred in reference areas, with Rs = 0.74 and 0.86 within the Middle Athabasca Region and SR, respectively. PPACs were more strongly associated with discharge than were APACs in the Middle Athabasca Region and the Lower Athabasca Region upstream of the AOSD (M0 PPAC Rs = 0.89, APAC Rs = 0.66; M2 PPAC Rs = 0.66, APAC Rs = 0.56). The reverse was true within and downstream of the AOSD, where APACs, which are prominent in the oil sands deposit, were more notably correlated with CQ (M3-M7 Rs = 0.68; M9 Rs = 0.41; M11a Rs = 0.84), suggesting potential PAC contributions from high discharge, erosion events. PPACs were not significantly correlated with CQ downstream of the AOSD.

**Fig. 5:**
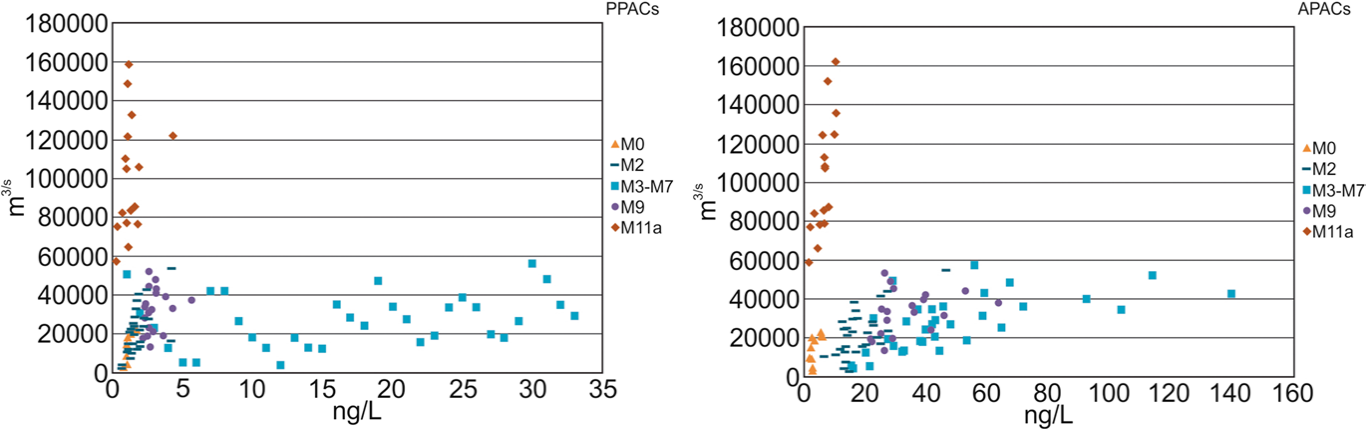
CQ versus PPAC and APAC concentrations in samples collected from the mainstem 2013–2019

PAC concentrations varied most notably within and between regions during the open-water season (Fig. 5). Under-ice concentrations clustered around the lower range of discharges. Within and immediately downstream the OSMA (M3-M7, M9) slight increases in discharge were associated with substantial increases in PACs, particularly the APACs. Discharges were generally lowest and least variable within the Middle Athabasca Region, typically falling below 20,000 m^3^/s. The highest occurred in the SR, exceeding those in all other regions; CQs for sampling events were between 57,530 m^3^/s and 158,630 m^3^/s. The range of discharges within the Lower Athabasca Region were relatively similar between regions, varying between 2528 m^3^/s and 56, 186 m^3^/s.

### Site-specific concentrations

**Fig. 6:**
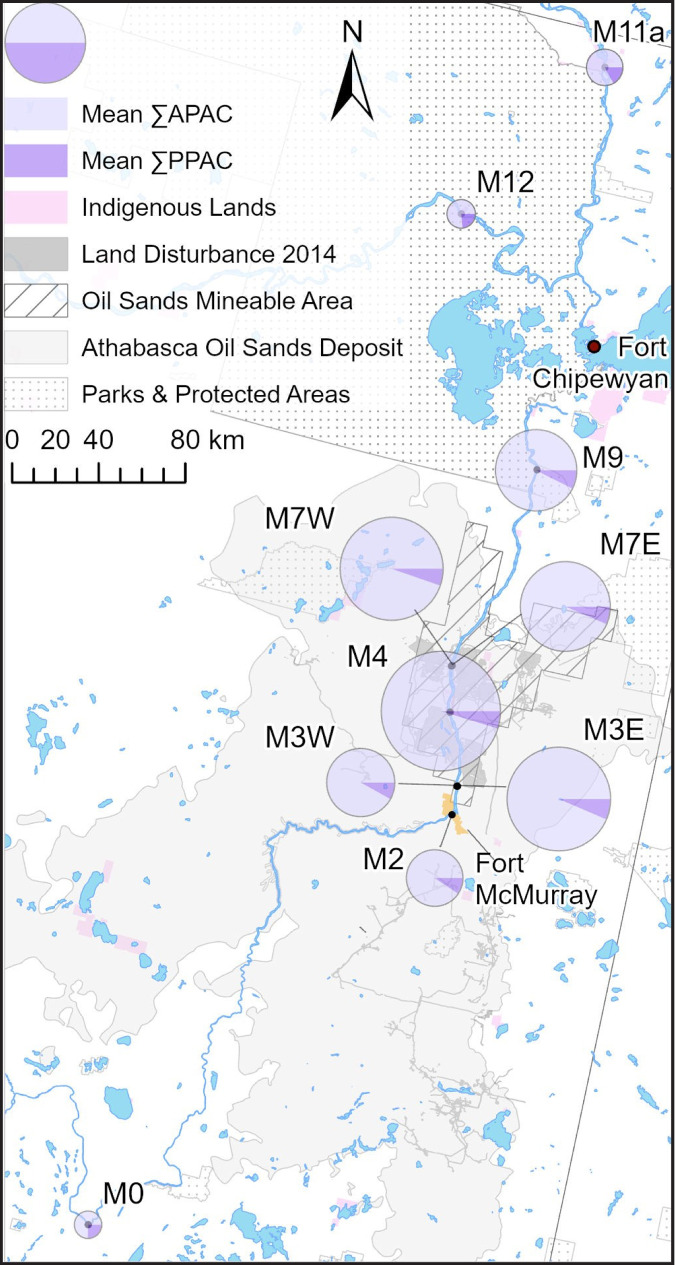
Mean site-specific PAC concentrations during the open-water season from 2013 to 2019

PAC concentrations along the AR mainstem changed along a gradient of exposure to the AOSD, tributary inflows, and anthropogenic activities. As seen with regional averages (Table 1), relative abundances (Fig.2), and interannual concentrations (Fig. 3), the lowest mean PAC concentrations from 2013 to 2019 occurred upstream and outside of the AOSD at M0, as well as downstream and outside of the Lower Athabasca Region (M11a on the SR, M12 on the PR; Fig. 6). Concentrations at these sites ranged between 1.22–1.46 ng/L PPAC and 3.63–7.12 ng/L APAC, with PPACs making up a higher proportion of TPACs than at sites within the AOSD and downstream on the lower AR. At these sites (M2, M3E, M3W, M4, M7E, M7W, M9), PPACs ranged between 1.94–4.92 ng/L and APACs between 18.7–84.5 ng/L.

**Fig. 7:**
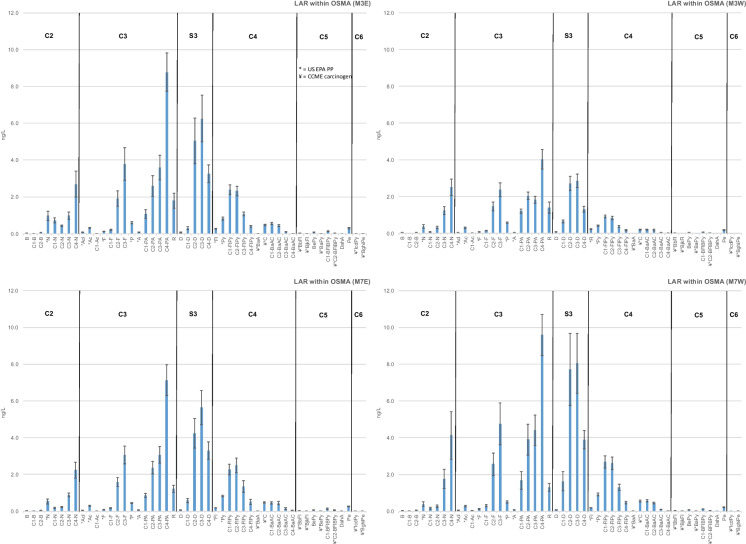
Mean relative abundances of PACs at M3E, M3W, M7E and M7W from 2015, 2017–2019

Mean PAC concentrations within the Lower Athabasca Region increased from the upstream-most extent of the AOSD (M2), reaching their highest near Fort McKay (M4), and falling slightly in concentration toward WBNP (M9; Fig. 6). Concentrations within the AOSD were at their lowest upstream of Fort McMurray (M2, 1.92 ng/L PPAC, 18.7 ng/L APAC). Downstream of Fort McMurray on the western side of the AR (M3W) concentrations increased only slightly to 2.42 ng/L PPAC and 27.4 ng/L APAC. In contrast, across the channel on the eastern side of the AR (M3E) downstream of the Clearwater, PPACs were over double those at M2, reaching 4.45 ng/L and APACs over triple at 64.7 ng/L. Further north into the OSMA at M4, downstream of the Steepbank River, mean PPAC concentrations remained similar to those at M3W (4.33 ng/L), while APAC concentrations increased by a third of those at M3W to a mean of 87.5 ng/L. Toward the southern-most extent of the OSMA concentrations decreased, with 3.33 ng/L PPAC and 48.7 ng/L APAC on the eastern side of the AR at M7E, and 3.62 ng/L PPAC and 64.6 ng/L APAC on the western side downstream of the Ells River (M7W). The increased PAC concentrations downstream of tributaries, particularly the Clearwater River, was also evident in the AOSD-characteristic profiles toward the east and west banks at M3 and M7 (Fig. 7). Of note is that, although concentrations differed, the profiles were similar at all four locations (M3E,W and M7E,W) regardless of primary water source. Outside of the AOSD at M9, PACs fell in concentration (PPAC 3.18 ng/L, APACs 39.4 ng/L), remaining above those measured at M3. This PAC gradient, with higher PAC concentrations within the AOSD downstream of anthropogenic activities and tributaries, was generally present in every monitoring year (Fig. SI4).

**Fig. 8:**
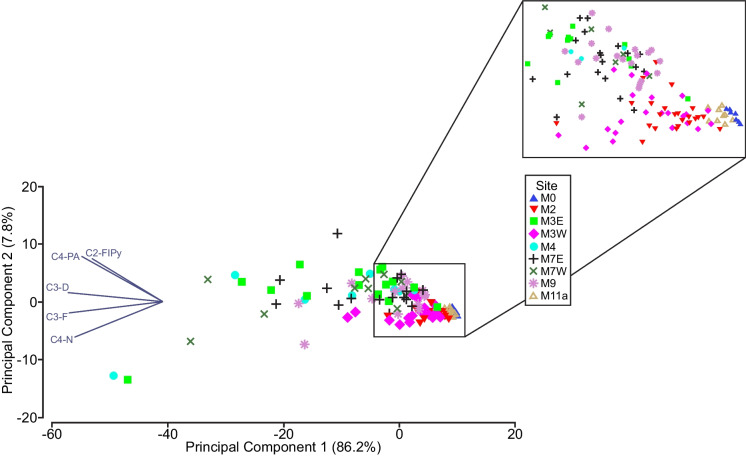
PCA for open-water samples collected within Middle Athabasca Region, Lower Athabasca Region, and SR from 2013 to 2019

**Fig. 9:**
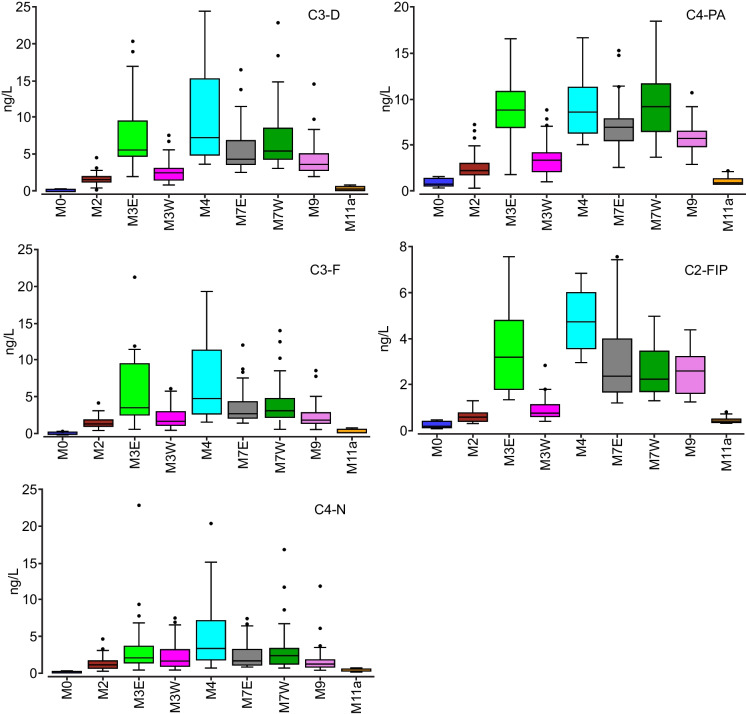
Mean site-specific concentrations of C3-D, C4-PA, C3-F, C2-FlPy and C4-N during the open-water season from 2013 to 2019

The PAC chemistry of sampling sites was most similar and least diverse for reference sites (M0, M11a), M2, and M3W (Fig. 8). These sites clustered tightly whereas sites within and downstream of the OSMA (M3E, M4, M7W, M7E, M9) varied more widely in chemistry, driven by concentrations of alkylated dibenzothiophenes (C2-C4) > phenanthrenes/anthracenes (C2-C4) > naphthalenes (C2, C3) > fluorenes (C2, C3) > fluoranthenes/pyrenes (C1, C2). Concentrations of C3-D, C4-PA, C3-F, C2-FlPy, and C4-N (Fig. 9) were highest in magnitude and variability within the OSMA (M3-M7) and downstream near WBNP (M9). The highest concentrations of these PACs were generally found at M4 upstream of Fort McKay, and downstream of the Clearwater and Steepbank rivers. This increase at M4 is particularly evident in C4-PA and C3-D, to a lesser degree in C3-FlPy and C3-Fl, and only slightly in C4-N. Concentrations of PACs remain relatively elevated at M9, similar to those near the downstream extent of the OSMA (M7E).

## Discussion

### Oil sands PACs in Athabasca mainstem water

Dissolved PAC concentrations in samples collected from the Lower Athabasca Region upstream, within and downstream of the OSMA exhibited relative abundances consistent with the AOSD. Alkylated PAs and Ds dominated, while alkylated Fs, Ns, FlPys and BaACs were present in more moderate quantities. This fingerprint was absent from the Middle Athabasca Region upstream of the AOSD and the PR located outside of the AR mainstem; most notably, these sites were distinguished by very low concentrations of C1-C4 Ds. Within the SR, at the downstream-most extent of the monitoring area, the AOSD fingerprint was depressed, with reduced relative abundance of Ds and absence of C1 < C2 < C3 < C4 abundances in alkylated homologs of N, F, PA and D. Substituted, petrogenic compounds, which have historically not been the focus of investigations into presence and effects of PACs in the environment (Andersson & Achten, 2015; Boehm & Saba, 2008; Jautzy et al., 2015), provide key information on the nature and presence of PACs upstream, within and downstream of the oil sands region.

Yang et al. (2011) first identified the chemical fingerprint of the AOSD from raw oil sands samples. They showed that characteristic relative abundances of alkylated compounds in these were P > D > F > C, with alkylated N concentrations exceeding those of F and C in one out of three samples. The abundance of homologs increased for those that were less easily biologically-degraded (C3 > C2 > C1 > C0). Samples of upgraded oil sands had higher relative abundances of Ns and Cs. The PIs of these samples were much higher than those for raw oil sands, reaching 0.12 as compared to 0.03 due to higher abundances of unsubstituted PACs in upgraded oil sands (Wang et al., 1999). In comparison, the PIs measured from samples collected within and downstream of the Lower Athabasca Region from 2013 to 2019 were for the most part between 0.03 and 0.1. These smaller unsubstituted:substituted ratios, as compared to those in raw oil sands samples, are consistent with the prominence of alkylated compounds in water of the AR.

Kelly et al. (2009) sampled snow and river water in 2008 along gradients of exposure to the McMurray Formation and oil sands development. Their results showed that airborne particulates contributed PAC loading to the snowpack; deposition decreased exponentially with distance from upgrading facilities up to a distance of 50 km. Snow particulates were relatively enriched in Ns and unsubstituted PACs, whereas the filtrate was dominated by dissolved Fls, Ds and PAs. Dissolved PAC concentrations in tributaries to the AR were dominated by alkylated Ds, PAs and Fls, followed by FlPys and BaACs, similar to the PACs measured in the Lower Athabasca Region for the OSM Program. The latter, however, had relatively higher abundances of Ns.

As part of OSM, Wang et al. (2014) conducted one of the most intensive multi-media studies of PACs in the oil sands region, analyzing hundreds of environmental samples collected between 2009 and 2012. River water, snowmelt water, and river sediment samples from the AR and its tributaries provided an indication of the differences between PACs present in each of these media, and their potential sources. Relative abundances of PACs from oil sands bitumen were similar to those found by Yang et al. (2011). Alkylated and unsubstituted PACs in water samples from the Athabasca, Steepbank, Ells and Firebag rivers, however, were for the most part below detection. Those in snowmelt samples, similar to PACs in the samples from Kelly et al. (2009), indicated that they originated from oil sands particulates in the vicinity of mining infrastructure and activity. Hydrocarbon profiles for sediments from the AR and tributaries suggested PACs originated from oil sands bitumen; however, the highest concentrations were measured in the Steepbank River, which at the time was the only tributary under development. The authors suggested that industrial activities could not be eliminated from consideration as potential contributors of PACs.

### Gradients in PAC concentrations

A clear gradient was present in the dissolved PAC chemistry of waters within the AR mainstem. Relative abundances of PACs within the Lower Athabasca Region were distinguished from those outside of, or distant from, the influence of the AOSD and OSMA (Middle Athabasca Region, PR, SR). Lower Athabasca Region samples had relatively higher concentrations of alkylated PAs, Ds, Ns, Fs, FlPys, and BaACs, increasing from C1 < C2 < C3 < C4, characteristic of the AOSD. This fingerprint was absent from M0 and M12, and depressed at M11a. PAC concentrations within the OSMA (M3, M4, M7) and downstream to the recovery site near WBNP (M9) were higher than those at the upstream and downstream reference sites (M0, M11a), within the AOSD upstream of the OSMA (M2), and outside of the AR mainstem on the PR (M12).

Dissolved PAC concentrations in the mainstem were related not only to the AOSD, but to tributary inflows and discharge. Inflows from the Clearwater and Ells rivers appeared to contribute to higher PAC concentrations in the mainstem at M3 and M7, particularly at M3E. PACs also increased with rising CQ, which may indicate contributions of PACs from channel erosion and surface water runoff from watersheds. Further downstream within the SR, lower magnitudes and differing relative abundances of PACs were attributed to buffering by the PAD and dilution by the PR. Elmes et al. (2016) concluded that retention and dilution likely caused the 45% reduction in concentrations of PACs deposited into the Slave River Delta (approximately 500 km downstream of the Alberta oil sands development and McMurray Formation), when compared to the Athabasca Delta upstream. Evans et al. (2002) found that surficial sediments and cores from water bodies including those in the PAD contained PACs in relative abundances similar to those reported herein. Hall et al. (2012) found alkylated homologs to be similar in flood deposits in the PAD and upstream in the oil sands region, pointing to downstream transport of naturally-occurring bituminous compounds.

Similar gradients in PAC concentrations within the AR and its tributaries have been reported by other studies and programs. Kelly et al. (2009) found that in 2008 dissolved PACs were higher in concentration downstream of oil sands development and associated with the proportion of the catchment within the McMurray Formation, overall land disturbance, and oil sands mining. The authors indicated that industrial PACs in snow particulates may have an effect on water bodies in close proximity to oil sands mining activities. Wang et al. (2014) also identified the potential for industrial contributions to water bodies, finding that PACs in sediments of the Ells, Firebag and, in particular, Steepbank rivers were greatest in their lower reaches within oil sands and mining intensive areas, versus their middle and upper reaches. Manzano et al. (2016), over 2 years of spring snowpack sampling from 2011 to 2014, showed that PAC concentration and deposition declined exponentially with distance from oil sands facilities; Pys, Cs and Ds dominated the distribution of these within the first 50 km. Mundy et al. (2019) found PAC concentrations in wetlands to be higher within 25 km of mining activities compared to farther afield. This was most notable for C2-,C3-, and C4-alkylated homologs, consistent with findings from the aforementioned snowmelt and sediment studies.

Studies have indicated the potential for effects of PACs on biota in and near the oil sands region. Wayland et al. (2008) in experimental and natural wetlands within the oil sands region showed that sediments and insects within wetlands preferentially accumulated alkylated PACs. Culp et al. (2018), as part of the long-term OSM benthic macroinvertebrate monitoring program, found higher concentrations of PACs in the lower reaches of the Steepbank and Ells rivers and associated changes in the composition of benthic assemblages, indicating mild environmental stress compared to upstream reaches. McMaster et al. (2018) fish monitoring on the Steepbank River showed that fish health (liver and reproductive energy) may be affected by PAC-related compounds in the downstream sections of the river (McMaster et al., 2018).

## Conclusions

Although work to date indicates that PACs in the environment within and downstream of the OSMA likely originate from natural sources, anthropogenic alterations to the lower AR watershed and climate must be considered in assessing the effects of the abundant, potentially toxic alkylated PACs in the region (Andersson & Achten, 2015; Culp et al., 2021). Culp et al. (2021), in integrating and synthesizing the datasets from seven OSM theme assessments, concluded that definitive association of oil sands activities with ecological effects (fish and benthic invertebrates) is confounded by inability at this time to differentiate between contaminants released due to oil sands operations and naturally-occurring compounds that originate from bitumen outcrops. This paper provides the first look at long-term, intensive information on baseline PAC concentrations within and downstream of the Athabasca River and explores potential regional influences related to oil sands mining and hydrology.

### Supplementary Information

ESM 1 (DOCX 314 kb)

## Data Availability

The datasets generated during and/or analyzed during the current study are available from the corresponding author on reasonable request.
