# Correction to: Dissolved polycyclic aromatic compounds in Canada’s Athabasca River in relation to Oil Sands from 2013 through 2019

**DOI:** 10.1007/s10661-024-12827-4

**Published:** 2024-06-24

**Authors:** Lucie M. J. Lévesque, Julie Roy, Nancy E. Glozier, Leah Dirk, Colin A. Cooke

**Affiliations:** 1 Environment and Climate Change Canada, Saskatoon, Saskatchewan S7N 3H5 Canada; 2 Environment and Parks, Government of Alberta, Edmonton, Alberta T5J 5C6 Canada; 3 Earth and Atmospheric Sciences, University of Alberta, Edmonton, Alberta T6G 2E3 Canada

**Correction to: Environ Monit Assess (2023) 195:1354**

10.1007/s10661-023-11846-x

The original version of this article unfortunately contained an error in Figure 1.

Corrected Figure 1 should have appeared as shown below.

**Fig. 1:**
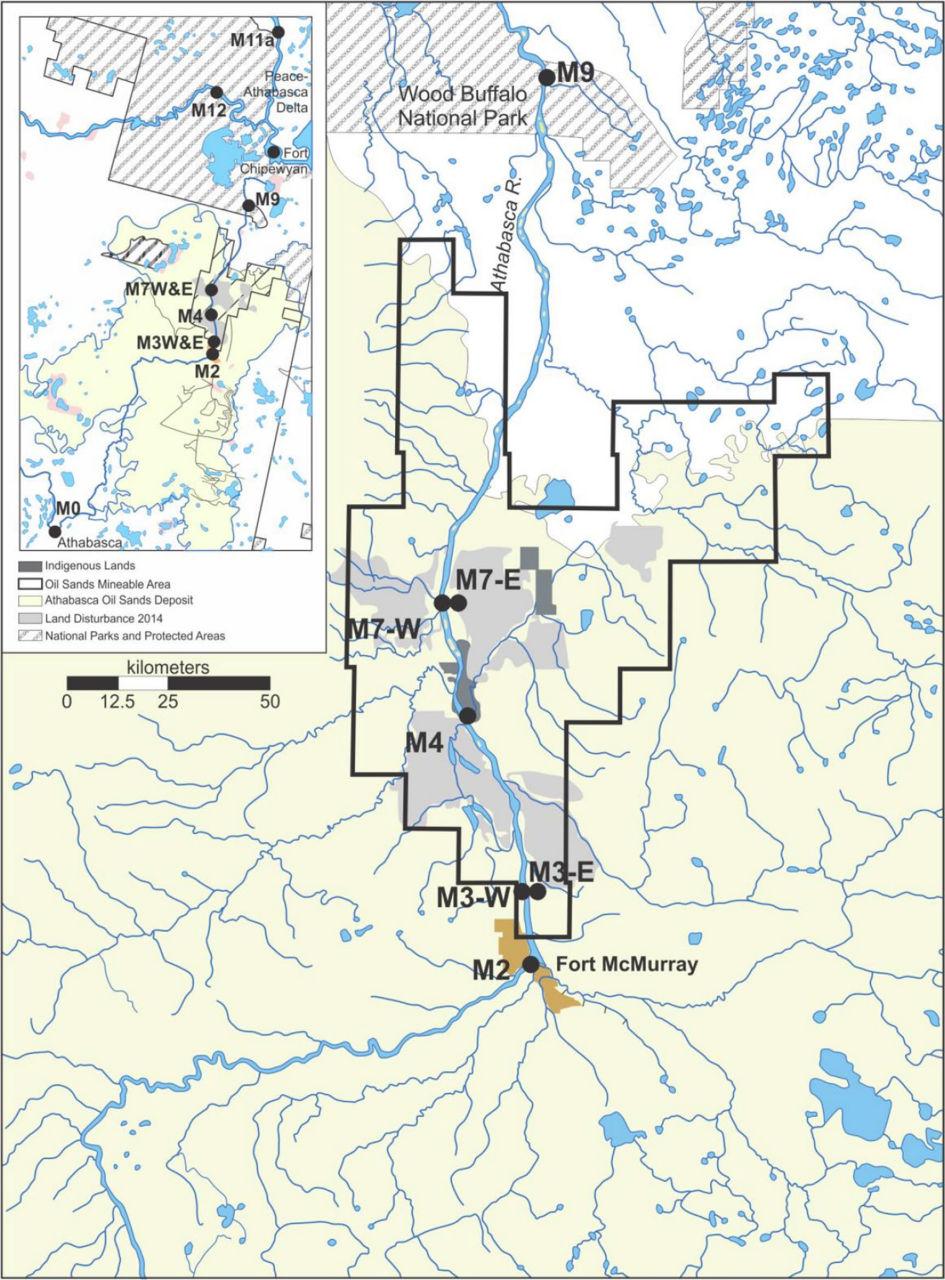
Monitoring area

