# Supplementary material for: Dissolved polycyclic aromatic compounds in Canada’s Athabasca River in relation to Oil Sands from 2013 through 2019
Source: Environ Monit Assess. 2023 Oct 21;195(11):1354. doi: 10.1007/s10661-023-11846-x (PMC10590302; doi:10.1007/s10661-023-11846-x)
Supplement: Supplementary file 1 — (DOCX 314 kb) [file 10661_2023_11846_MOESM1_ESM.docx]

**Title**

Dissolved Polycyclic Aromatic Compounds in Canada’s Athabasca River in Relation to Oil Sands from 2013 through 2019: Supplementary Information

**Authors**

Lucie M.J. Lévesque^1*^, Julie Roy^1^, Nancy E. Glozier^1^ , Leah Dirk^1^, Colin A. Cooke^2,3^

^1^Environment and Climate Change Canada, Saskatoon, Saskatchewan, S7N 3H5

^2^Environment and Parks, Government of Alberta, Edmonton, Alberta T5J 5C6, Canada

^3^Earth and Atmospheric Sciences, University of Alberta, Edmonton, Alberta T6G 2E3, Canada

[^*^lucie.levesque@ec.gc.ca](mailto:*lucie.levesque@ec.gc.ca), (604) 644-9345


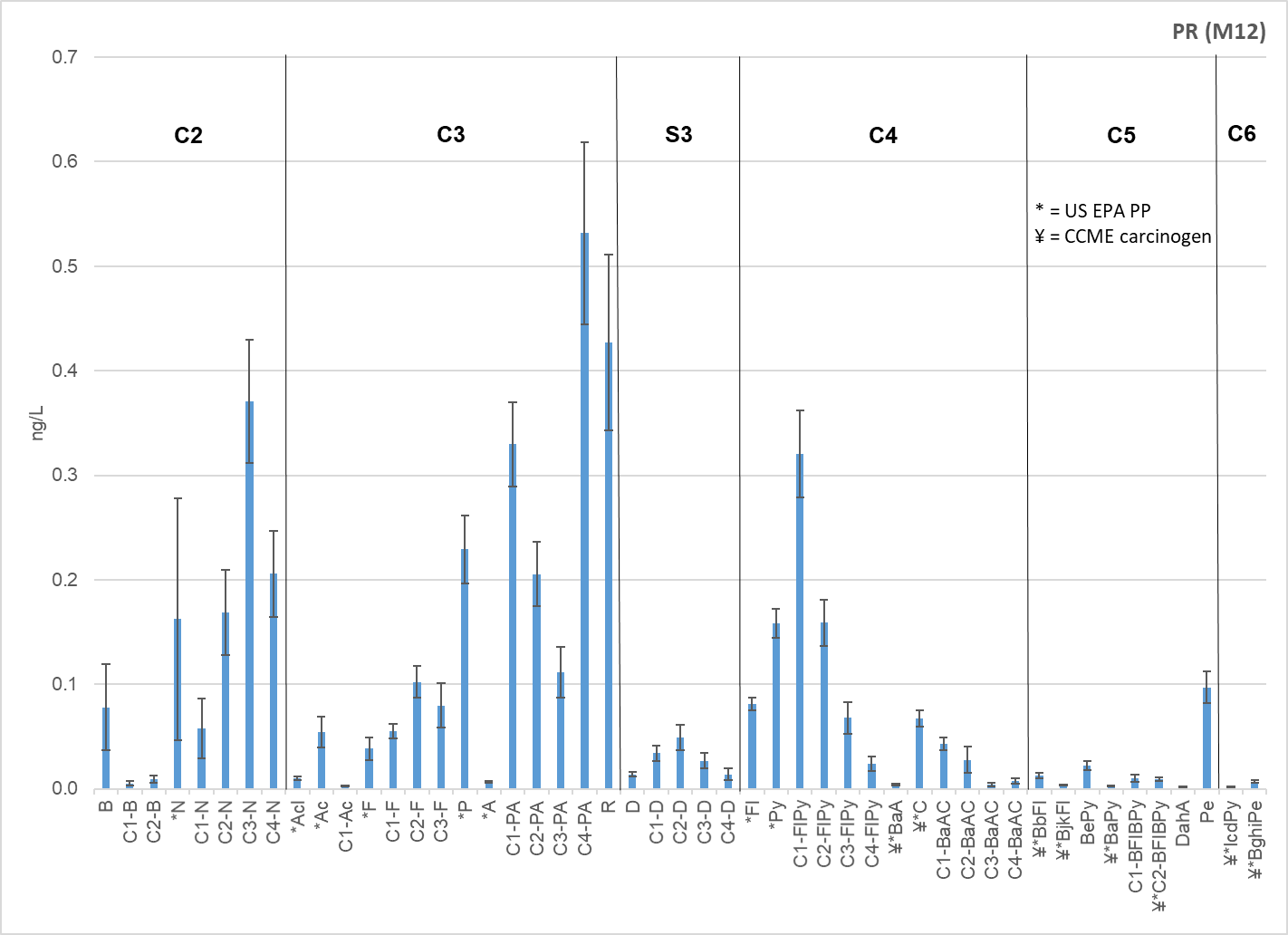
**Table SI1** Samples collected from the Peace River (PR) at Peace Point (M12) from 2013-2019.

| **YEAR** | **PR** |
| --- | --- |
| **2013** | M12^(2)^ |
| **2014** | M12^(2,4)^ |
| **2015** | M12^(1,4)^ |
| **2016** | n/s |
| **2017** | n/s |
| **2018** | n/s |
| **2019** | n/s |

n/s = no samples; (N) = number of open-water samples; (N, N) = number of under-ice, open-water samples

**Fig. SI1** Mean 2013-2019 relative abundances of PACs within the PR

**Table SI2.** SGS AXYS Analytical Services Ltd. – SOP MLA-021 Rev.12, Version 9 – “Analytical Method for the Determination of Polycyclic Aromatic Hydrocarbons (PAH), Alkylated PAHs, and Alkanes. Appendix 4 Excerpts: Quantification of SPMD and PUF Samples with PRC (Client, Field, and Photolysis Standards): details target compounds, surrogates by purpose, and quantification references for SPMD and PUF samples where PRC surrogates PAH compounds are used. The standard SPMD design is to use d10 anthracene and d10- fluoranthene as PRC compounds, and d14 – dibenz(a,h)anthracene as a photolytic standard. The default quantification scheme is modified as follows to accommodate the re-purposing of these compounds.

SPMD Matrix - Analyte Ions Monitored, Surrogates Used and RRF Determination for PAH using Client, Field, PRC and Photolysis Standards.

| **TARGET ANALYTES** | **Quantification**  **Ion (m/z)** | **SURROGATE** | **RRF DETERMINED FROM** |
| --- | --- | --- | --- |
| Naphthalene | 128 | d_8_-Naphthalene | Naphthalene |
| Acenaphthylene | 152 | d_8_-Acenaphthylene | Acenaphthylene |
| Acenaphthene | 154 | d_8_-Acenaphthylene | Acenaphthene |
| Fluorene | 166 | d_10_-Phenanthrene | Fluorene |
| Phenanthrene | 178 | d_10_-Phenanthrene | Phenanthrene |
| Anthracene | 178 | d_10_-Phenanthrene | Anthracene |
| Fluoranthene | 202 | d_10_-Phenanthrene | Fluoranthene |
| Pyrene | 202 | d_10_-Phenanthrene | Pyrene |
| Benz[a]anthracene ^6^ | 228 | d_12_-Benz[a]anthracene | Benz[a]anthracene |
| Chrysene ^1^ | 228 | d_12_-Chrysene | Chrysene |
| Benzo[b]fluoranthene | 252 | d_12_-Benzo[b]fluoranthene | Benzo[b]fluoranthene |
| Benzo[j,k]fluoranthenes | 252 | d_12_-Benzo[k]fluoranthene | Benzo[k]fluoranthene |
| Benzo[e]pyrene | 252 | d_12_-Benzo[a]pyrene | Benzo[e]pyrene |
| Benzo[a]pyrene | 252 | d_12_-Benzo[a]pyrene | Benzo[a]pyrene |
| Perylene | 252 | d_12_-Perylene | Perylene |
| Dibenzo[ah]anthracene ^2^ | 278 | d_12_-Indeno[1,2,3,cd]pyrene | Dibenz[ah]anthracene |
| Indeno[1,2,3-cd]pyrene | 276 | d_12_-Indeno[1,2,3,cd]pyrene | Indeno[1,2,3-cd]pyrene |
| Benzo[ghi]perylene | 276 | d_12_-Benzo[ghi]perylene | Benzo[ghi]perylene |
| Biphenyl ^3^ | 154 | d_10_- Biphenyl | Biphenyl |
| Dibenzothiophene ^3^ | 184 | d_8_-Dibenzothiophene | Dibenzothiophene |
| 1-Methylnaphthalene ^3^ | 142 | d_10_-2-Methylnaphthalene | 1-Methylnaphthalene |
| 2-Methylnaphthalene ^3^ | 142 | d_10_-2-Methylnaphthalene | 2-Methylnaphthalene |
| C1-Naphthalenes ^3^ | 142 | d_10_-2-Methylnaphthalene | 1- & 2-Methylnaphthalene |
| 2,6-Dimethylnaphthalene ^3^ | 156 | d_12_-2,6 Dimethylnaphthalene | 2,6-Dimethylnaphthalene |
| 1,2-Dimethylnaphthalene | 156 | d_12_-2,6 Dimethylnaphthalene | 1,2-Dimethylnaphthalene |
| C2-Naphthalenes ^3^ | 156 | d_12_-2,6 Dimethylnaphthalene | 2,6- & 1,2-Dimethylnaphthalene |
| 2,3,5-Trimethylnaphthalene ^3^ | 170 | d_12_-2,6 Dimethylnaphthalene | 2,3,5- Trimethylnaphthalene |
| 2,3,6-Trimethylnaphthalene | 170 | d_12_-2,6 Dimethylnaphthalene | 2,3,6- Trimethylnaphthalene |
| C3-Naphthalenes ^3^ | 170 | d_12_-2,6 Dimethylnaphthalene | 2,3,5- & 2,3,6-Trimethylnaphthalene |
| 1,4,6,7-Tetramethylnaphthalene | 184 | d_12_-2,6 Dimethylnaphthalene | 1,4,6,7-Tetramethylnaphthalene |
| C4-Naphthalene | 184 | d_12_-2,6 Dimethylnaphthalene | 1,4,6,7-Tetramethylnaphthalene |
| 2-Methylanthracene | 192 | d_10_-Phenanthrene | 2-Methylanthracene |
| 3-Methylphenanthrene | 192 | d_10_-Phenanthrene | 1- & 2-Methylphenanthrene & 2-Methylanthracene |
| 2-Methylphenanthrene | 192 | d_10_-Phenanthrene | 2-Methylphenanthrene |
| 9/4-Methylphenanthrenes | 192 | d_10_-Phenanthrene | 1- & 2-Methylphenanthrene & 2-Methylanthracene |
| 1-Methylphenanthrene ^3^ | 192 | d_10_-Phenanthrene | 1-Methylphenanthrene |
| C1-Phenanthrenes/Anthracenes ^3^ | 192 | d_10_-Phenanthrene | 1- & 2-Methylphenanthrene & 2-Methylanthracene |
| 3,6-Dimethylphenanthrene ^3^ | 206 | d_10_-Phenanthrene | 3,6-Dimethylphenanthrene |
| 2,6-Dimethylphenanthrene | 206 | d_10_-Phenanthrene | 3,6- & 1,7-Dimethyl­phenanthrenes |
| 1,7-Dimethylphenanthrene | 206 | d_10_-Phenanthrene | 1,7-Dimethylphenanthrene |
| 1,8-Dimethylphenanthrene | 206 | d_10_-Phenanthrene | 3,6- & 1,7-Dimethyl­phenanthrenes |
| C2-Phenanthrenes/Anthracenes ^3^ | 206 | d_10_-Phenanthrene | 3,6- & 1,7-Dimethyl­phenanthrenes |
| 1,2,6-Trimethylphenanthrene | 220 | d_10_-Phenanthrene | 1,2,6-Trimethylphenanthrene |
| C3-Phenanthrenes/Anthracenes | 220 | d_10_-Phenanthrene | 1,2,6-Trimethylphenanthrene |
| Retene ^3^ | 234 | d_10_-Phenanthrene | Retene |
| C4-Phenanthrenes/Anthracenes | 234 | d_10_-Phenanthrene | Retene |
| C1-Biphenyls | 168 | d_10_- Biphenyl | Biphenyl |
| C2-Biphenyls | 182 | d_10_- Biphenyl | Biphenyl |
| C1-Acenaphthenes | 168 | d_8_-Acenaphthylene | Acenaphthene |
| 2-Methylfluorene | 180 | d_10_-Phenanthrene | 2-Methylfluorene |
| C1-Fluorenes | 180 | d_10_-Phenanthrene | 2-Methylfluorene |
| 1,7-Dimethylfluorene | 194 | d_10_-Phenanthrene | 1,7-Dimethylfluorene |
| C2-Fluorenes | 194 | d_10_-Phenanthrene | 1,7-Dimethylfluorene |
| C3-Fluorenes | 208 | d_10_-Phenanthrene | 1,7-Dimethylfluorene |
| 2/3-Methyldibenzothiophenes | 198 | d_8_-Dibenzothiophene | 2/3-Methyldibenzothiophenes |
| C1-Dibenzothiophenes | 198 | d_8_-Dibenzothiophene | 2/3-Methyldibenzothiophenes |
| 2,4-Dimethyldibenzothiophene | 212 | d_8_-Dibenzothiophene | 2,4-Dimethyldibenzothiophene |
| C2-Dibenzothiophenes | 212 | d_8_-Dibenzothiophene | 2,4-Dimethyldibenzothiophene |
| C3-Dibenzothiophenes | 226 | d_8_-Dibenzothiophene | 2,4-Dimethyldibenzothiophene |
| C4-Dibenzothiophenes | 240 | d_8_-Dibenzothiophene | 2,4-Dimethyldibenzothiophene |
| 3-Methylfluoranthene/Benzo[a]fluorene | 216 | d_10_-Phenanthrene | 3-Methylfluoranthene |
| C1-Fluoranthenes/Pyrenes | 216 | d_10_-Phenanthrene | 3-Methylfluoranthene |
| C2-Fluoranthenes/Pyrenes | 230 | d_10_-Phenanthrene | 3-Methylfluoranthene |
| C3-Fluoranthenes/Pyrenes | 244 | d_10_-Phenanthrene | 3-Methylfluoranthene |
| C4-Fluoranthenes/Pyrenes | 258 | d_10_-Phenanthrene | 3-Methylfluoranthene |
| 5/6-Methylchrysenes | 242 | d_12_-Chrysene | 6-Methylchrysene |
| 1-Methylchrysene | 242 | d_12_-Chrysene | 1-Methylchrysene |
| C1-Benzo[a]anthracenes/Chrysenes | 242 | d_12_-Chrysene | 1- & 6-Methylchrysenes |
| 5,9-Dimethylchrysene | 256 | d_12_-Chrysene | 5,9-Dimethylchrysene |
| C2-Benzo[a]anthracenes/Chrysenes | 256 | d_12_-Chrysene | 5,9-Dimethylchrysene |
| C3-Benzo[a]anthracenes/Chrysenes | 270 | d_12_-Chrysene | 5,9-Dimethylchrysene |
| C4-Benzo[a]anthracenes/Chrysenes | 284 | d_12_-Chrysene | 5,9-Dimethylchrysene |
| 7-Methylbenzo[a]pyrene | 266 | d_12_-Benzo[a]pyrene | 7-Methylbenzo[a]pyrene |
| C1-Benzofluoranthenes/Benzo­pyrenes | 266 | d_12_-Benzo[a]pyrene | 7-Methylbenzo[a]pyrene |
| C2-Benzofluoranthenes/Benzo­pyrenes | 280 | d_12_-Benzo[a]pyrene | 7-Methylbenzo[a]pyrene |
| **LABELLED CLIENT/FIELD STANDARDS** | **Quantification Ion (m/z)** | **RECOVERY CALCULATED AGAINST** | **RRF DETERMINED FROM** |
| d_10_-Anthracene | 188 | d_10_-Phenanthrene |  |
| d_10_-Fluoranthene | 212 | d_12_-Benzo[a]anthracene |  |
| **LABELLED PHOTOLYSIS STANDARD** | **Quantification Ion (m/z)** | **RECOVERY CALCULATED AGAINST** | **RRF DETERMINED FROM** |
| d_14_-Dibenzo[ah]anthracene | 292 | d_12_-Indeno[1,2,3-cd]pyrene |  |
| **LABELLED SURROGATE STANDARDS** | **Quantification Ion (m/z)** | **RECOVERY CALCULATED AGAINST** |  |
| d_8_-Naphthalene | 136 | d_10_-Acenaphthene |  |
| d_10_-2-Methylnaphthalene | 152 | d_10_-Acenaphthene |  |
| d_10_-Biphenyl | 164 | d_10_-Acenaphthene |  |
| d_12_-2,6-Dimethylnaphthalene | 168 | d_10_-Acenaphthene |  |
| d_8_-Acenaphthylene | 160 | d_10_-Acenaphthene |  |
| d_8_-Dibenzothiophene | 192 | d_10_-Pyrene |  |
| d_10_-Phenanthrene | 188 | d_10_-Pyrene |  |
| d_12_-Benz[a]anthracene | 240 | d_10_-Pyrene |  |
| d_12_-Chrysene | 240 | d_10_-Pyrene |  |
| d_12_-Benzo[b]fluoranthene | 264 | d_12_-Benzo[e]pyrene |  |
| d_12_-Benzo[k]fluoranthene | 264 | d_12_-Benzo[e]pyrene |  |
| d_12_-Benzo[a]pyrene | 264 | d_12_-Benzo[e]pyrene |  |
| d_12_-Perylene | 264 | d_12_-Benzo[e]pyrene |  |
| d_12_-Indeno[1,2,3,cd]pyrene | 288 | d_12_-Benzo[e]pyrene |  |
| d_12_-Benzo[ghi]perylene | 288 | d_12_-Benzo[e]pyrene |  |

**Table SI3** PAC concentrations in the Middle Athabasca Region, Lower Athabasca Region upstream of (M2), within (M3-M7) and downstream of (M9) the OSMA, the SR, and the PR from 2013-2019

| **Region within Monitoring Area** | **Middle Athabasca Region (MAR)** | | | **Lower Athabasca Region (LAR)** | | | | | | | | | **Slave River (SR)** | | | **Peace River (PR)** | | |
| --- | --- | --- | --- | --- | --- | --- | --- | --- | --- | --- | --- | --- | --- | --- | --- | --- | --- | --- |
| **Site ^(N)^** | **M0 ^(11)^** | | | **M2 ^(30)^** | | | **M3-M7^(101)^** | | | **M9^(27)^** | | | **M11a^(16)^** | | | **M12^(13)^** | | |
| PAC Subset | Mean ng/L | SE | %ND/  Sample | Mean ng/L | SE | %ND/  Sample | Mean ng/L | SE | %ND/  Sample | Mean ng/L | SE | %ND/  Sample | Mean ng/L | SE | %ND/  Sample | Mean ng/L | SE | %ND/  Sample |
| ∑PACs (TPAC) | 4.66 | 0.50 | 2-37 | 19.98 | 1.49 | 0-39 | 52.80 | 4.03 | 0-39 | 41.62 | 4.16 | 2-39 | 7.78 | 0.83 | 10-45 | 4.51 | 0.62 | 12-55 |
| ∑Parent (PPAC) | 1.17 | 0.08 | 0-53 | 1.80 | 0.16 | 0-53 | 3.27 | 0.17 | 0-53 | 3.10 | 0.23 | 5-53 | 1.31 | 0.23 | 11-68 | 1.05 | 0.08 | 11-68 |
| ∑Alkylated (APAC) | 3.49 | 0.44 | 0-37 | 18.18 | 1.37 | 0-30 | 49.53 | 3.90 | 0-30 | 38.51 | 3.98 | 0-30 | 6.46 | 0.67 | 7-33 | 3.46 | 0.02 | 3-47 |
| ∑EPA 16 PP | 1.02 | 0.05 | 0-56 | 1.56 | 0.14 | 0-63 | 2.86 | 0.15 | 0-56 | 2.67 | 0.22 | 0-56 | 1.07 | 0.20 | 13-69 | 0.84 | 0.05 | 13-75 |
| ∑CCME | 0.09 | 0.01 | 0-88 | 0.19 | 0.01 | 0-75 | 0.57 | 0.03 | 0-88 | 0.61 | 0.03 | 0-63 | 0.17 | 0.02 | 0-88 | 0.10 | 0.01 | 13-88 |
| ∑C2 | 0.45 | 0.08 | 0-75 | 2.51 | 0.32 | 0-75 | 5.48 | 0.58 | 0-75 | 3.62 | 0.86 | 0-3 | 1.15 | 0.26 | 0-75 | 1.06 | 0.08 | 0-63 |
| ∑C3 | 2.83 | 0.26 | 0-14 | 10.01 | 0.83 | 0-36 | 22.87 | 1.73 | 0-36 | 16.45 | 1.69 | 0-43 | 3.60 | 0.45 | 7-43 | 2.18 | 0.26 | 7-43 |
| ∑S3 | 0.16 | 0.05 | 0-40 | 4.60 | 0.38 | 0-20 | 14.77 | 1.33 | 0-40 | 11.19 | 1.43 | 0-40 | 0.87 | 0.15 | 0-40 | 0.14 | 0.05 | 0-60 |
| ∑C4 | 1.05 | 0.18 | 0-33 | 2.59 | 0.16 | 0-25 | 9.10 | 0.59 | 0-25 | 9.71 | 0.65 | 0-25 | 1.89 | 0.17 | 0-25 | 0.97 | 0.11 | 0-50 |
| ∑C5 | 0.16 | 0.04 | 0-88 | 0.25 | 0.03 | 0-63 | 0.54 | 0.03 | 0-88 | 0.61 | 0.03 | 0-63 | 0.25 | 0.03 | 0-63 | 0.16 | 0.02 | 13-88 |
| ∑C6 | 0.01 | 0.00 | 0-100 | 0.01 | 0.00 | 0-100 | 0.02 | 0.00 | 0-100 | 0.03 | 0.00 | 0-59 | 0.01 | 0.00 | 0-11 | 0.01 | 0.00 | 0-100 |

N = number of samples; TPAC = total PACs (sum of 49 analytes) per sample; PPAC = parent (unsubstituted) PACs; APAC = alkylated PACs; EPA PP = (United States) Environmental Protection Agency (unsubstituted) Priority Pollutants; CCME = Canadian Council of Ministers of the Environment carcinogenic (unsubstituted) PACs

**Fig. SI2** PPAC and APAC concentrations during open-water season in the Middle Athabasca Region (M0), Lower Athabasca Region (M2, M3-M7, M9) and the SR (M11a) from 2013-2019. Median plotted with 10th, 25th, 75th, and 90th percentiles.

**Fig. SI2-i** TPAC concentrations during under-ice season 2014-2015. Median plotted with 10th, 25th, 75th, and 90th percentiles.

**Fig. SI3** Retene (R) during open-water season in the Middle Athabasca Region (M0), Lower Athabasca Region (M2, M3-M7, M9) and the SR (M11a) from 2013-2019. Median plotted with 10th, 25th, 75th, and 90th percentiles.

**Table SI4** Relationships between PAC concentrations and CQ

| **CQ vs PACS** | | **MAR** | **LAR** | | | **SR** |
| --- | --- | --- | --- | --- | --- | --- |
|  |  | **M0** | **M2** | **M3-M7** | **M9** | **M11a** |
| **WSC Station** | | **07BE001** | **07DA001 less 07CD001** | **07DA001** | **07DD001** | **07NB001** |
| **TPAC** | **Rs (p<0.05)** | 0.74  (8.1x10^-3^) | 0.62  (2.8x10^-4^) | 0.68  (8.9x10^-6^) | 0.36  (0.12) | 0.86  (2.0x10^-7^) |
| **APAC** | **Rs (p<0.05)** | 0.66  (2.3x10^-2^) | 0.56  (1.3x10^-3^) | 0.68  (8.3x10^-6^) | 0.41  (7.9x10^-2^) | 0.84  (2.0x10^-7^) |
| **PPAC** | **Rs (p<0.05)** | 0.89  (2.0x10^-7^) | 0.66  (5.3x10^-5^) | 0.62  (1.1x10^-4^) | 0.28  (0.24) | 0.40  (0.12) |

^CQ = cumulative discharge; Rs = Spearman Rank correlation coefficient; N = number of samples; p = probability; - = correlation not significant at 95%.^

**Fig. SI4** Mean PACs during the open-water season at all sites from upstream to downstream within the monitoring area from 2013-2019.

*
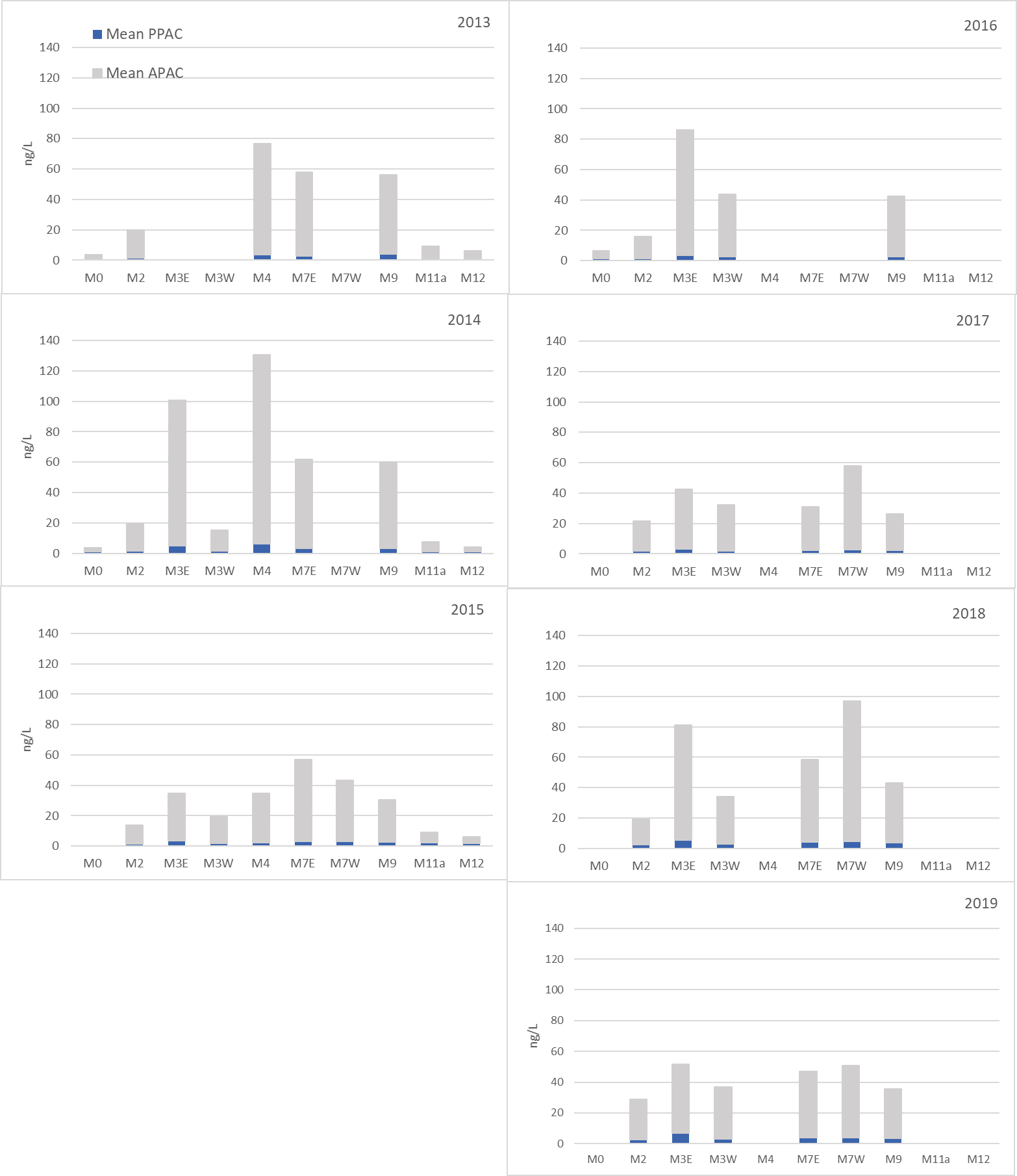
*
